# Association of *β*2-Microglobulin with Physical Performance in Chinese Hemodialysis Patients with and without Diabetes

**DOI:** 10.34067/KID.0000000669

**Published:** 2024-12-03

**Authors:** Qiu-Nan Zhan, Wen-Wen Chu, Qi Guo, Jun-Li Zhao

**Affiliations:** 1 Department of Nephrology, Shanghai University of Medicine and Health Sciences Affiliated Zhoupu Hospital, Shanghai, China; 2 Department of Rehabilitation Medicine, Shanghai University of Medicine and Health Sciences Affiliated Zhoupu Hospital, Shanghai, China

**Keywords:** chronic renal failure, diabetes, dialysis, hemodialysis

## Abstract

**Key Points:**

- In the patients on hemodialysis, *β*2-microglobulin was lower in those with worse physical performance than in those with better physical performance.
- A significant association of higher *β*2-microglobulin was discovered with better physical performance in the patients on hemodialysis without diabetes.

**Background:**

Higher *β*2-microglobulin (*β*2-MG) is associated with aging, stroke, and cognitive impairment, which are all connected with poor physical fitness. Poor physical function is ascribed to increasing mortality. However, there has been an academic dispute over the association of serum *β*2-MG with survival rate. Furthermore, diabetes mellitus (DM) has been well linked to poor physical function and a high level of *β*2-MG. We hypothesized that higher *β*2-MG could be associated with worse physical performance in patients on hemodialysis and that this association could vary with diabetes.

**Methods:**

We conducted a multicenter cross-sectional study at seven hemodialysis centers in Shanghai and Suzhou in China, where a collection was made of the clinical characteristics, laboratory indicators, physical performance, and DM assessment. Physical function was measured by the Short Physical Performance Battery (SPPB), and SPPB score ≤9 was considered as the cutoff for low physical performance. The patients were divided into two groups of low and high physical performance before being categorized into four subgroups on the basis of the absence of diabetes and SPPB scores. Logistic regression analyses were conducted to explore the association of *β*2-MG with physical performance.

**Results:**

The final analysis involved a total of 780 patients, 251 (32.2%) having diabetes. In the total population, *β*2-MG was lower in those with low SPPB scores than in those with high SPPB scores. Only in nondiabetic patients, *β*2-MG was lower in the low-SPPB group and positively associated with SPPB and its three component scores. Regardless of diabetes status, those who had low SPPB scores were prone to be older, accompanied by poor nutritional status. In addition, diabetic patients tended to have shorter hemodialysis duration and higher body mass index than the nondiabetic patients. Both before and after the covariates were adjusted, *β*2-MG was significantly associated with physical performance in patients on hemodialysis without diabetes.

**Conclusions:**

Lower levels of *β*2-MG were significantly associated with poor physical performance in Chinese patients on hemodialysis without DM.

## Visual Abstract

**Figure:**
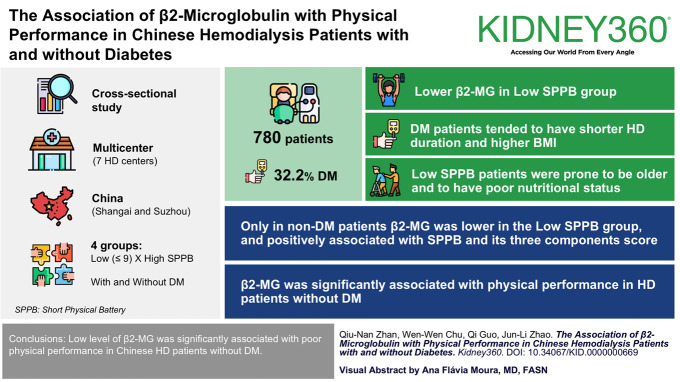

## Introduction

Given the increasing prevalence of ESKD and its associated burden on health, it is urgent to improve the clinical outcomes and quality of life of patients with ESKD.^1^ It is well recognized that patients with CKD, especially those undergoing hemodialysis, have poor physical performance.^2,3^

*β*2-microglobulin (*β*2-MG) is an 11.8 kDa protein produced by all cells that express major histocompatibility class 1 genes. Most of *β*2-MG is normally eliminated by the kidney by glomerular filtration and subsequent tubular catabolism. In the presence of renal dysfunction, serum *β*2-MG concentrations increase progressively. The *β*2-MG has long been used as a marker of uremic toxins in the middle molecule range, particularly for the assessment of dialysis adequacy.^4^ Higher *β*2-MG has been proven to be associated with aging, stroke, and cognitive impairment,^5,6^ which are all associated with poor physical fitness.^7–9^ It has been verified that poor physical function can result in poor quality of life, increasing morbidity and mortality.^10–12^ However, there has been a dispute over the association of serum *β*2-MG and survival; some previously reported studies demonstrated that elevated *β*2-MG was associated with better survival in patients on dialysis,^13,14^ while other reported the conclusions in the opposite direction.^15–17^ Thus, the association of *β*2-MG with physical fitness remains unclear. It was reported that diabetes was associated with risk of poor physical function in patients on dialysis.^18^ Our previous study demonstrated that poor physical performance was associated with mild cognitive impairment in the diabetic hemodialysis group rather than in the nondiabetic hemodialysis group.^19^ Furthermore, the elevated glucose concentrations were positively associated with *β*2-MG concentrations in patients on dialysis with or without diabetes.^20^ In view of this, we hypothesized that higher *β*2-MG was associated with worse physical performance in patients on hemodialysis and that the association of *β*2-MG and physical performance with diabetes could differ from that without diabetes. Therefore, we conducted a multicenter study to explore the association of *β*2-MG and physical performance in Chinese patients on hemodialysis with and without diabetes.

## Methods

### Recruitment

This study was part of a multicenter cross-sectional study conducted at seven hemodialysis centers in Shanghai and Suzhou in China (ChiCTR1900027039) between July 2020 and April 2021. From each center, the data were collected using a strict quality control framework. Patients were eligible to participate if they were older than 18 years and were on hemodialysis for 4 hours per session, twice or three times a week, for >3 months. For hemodialysis, the vascular access modalities involved fistulas and catheters, and dialyzer models included F14, LOPS15, FX80, *etc*.

Patients were excluded because of the following criteria: (*1*) inability to communicate with interviewers; (*2*) *β*2-MG data lost; (*3*) inability to complete the performance-based tests; (*4*) hearing/visual impairment; and (*5*) a severe infection, amputated limb, malignancy, or serious illness interfering with the conduct of the study or interpretation of the results.

In the study, approved by the Ethics Committee of Shanghai University of Medicine and Health Sciences (number 2019-A4-2621-19-201001-03-12010419771113601X), the methods were conducted following the principles of the Declaration of Helsinki. Verbal informed consent was obtained from all patients before enrollment.

### Clinical Characteristics

The patients' demographics and comorbidities were recorded, which referred to age, sex, postdialysis body mass index (BMI), hemodialysis duration, hypertension and diabetes, and a history of cardiovascular disease (CVD). CVD was recorded if one of the following conditions was present: angina, class 3–4 congestive heart failure (New York Heart Association), transient ischemic attack, history of myocardial infarction or cerebrovascular accident, or peripheral arterial disease. Behavioral characteristics were referred to as smoking and drinking habits. Nutritional status was assessed by Malnutrition Inflammation Score (MIS).^21^ Comorbidity was assessed by Charlson Comorbidity Index (CCI), which accounted for multiple comorbidities by creating a sum score according to the presence of 19 comorbid conditions.^22^ Muscle mass was measured using a direct segmental multifrequency bioelectrical impedance analysis (six In-Body 720; Biospace Co. Ltd, Seoul, Korea). According to the Asian Working Group for Sarcopenia criteria,^23^ low muscle mass was classified as relative skeletal muscle mass index (the appendicular skeletal muscle mass (ASM)/ height^2^) <7.0 and 5.7 kg/m^2^ in men and women, respectively.

### Laboratory Measures

Biochemical data, collected before dialysis, referred to hemoglobin, serum albumin, serum creatinine, BUN, uric acid, calcium, phosphate, intact parathyroid hormone (iPTH), *β*2-MG, C-reactive protein, and lipid profiles within 3 months of physical assessment. Dialysis adequacy is defined by Kt/V and urea reduction ratio (URR). Kt/V was used as an indicator of dialysis quality and monitored in every dialysis treatment using the Online Clearance Monitor (Fresenius Medical Care, Bad Homburg, Germany).

### Performance-Based Assessment

Physical performance was measured with the Short Physical Performance Battery (SPPB),^24^ where the participants were asked to perform three timed tasks: the standing balance test, the five-repetition sit-to-stand test, and the 4-m gait speed test. The standing balance test required participants to maintain each of the positions for 10 seconds as follows: standing with the feet in the side-by-side position, semitandem position, and tandem position, respectively. The five-repetition sit-to-stand test required participants to stand up and sit down five times as quickly as possible with their hands folded across their chest. The 4-m gait speed test measured the time required for walking 4 meters at the usual pace. This test was repeated twice, the shorter time of the two measurements used for analysis. The timed results of each subtest were rescaled according to the predefined cut points for obtaining a performance score ranging from 0 (worst) to 4 (best). The total score of SPPB ranges from 0 to 12. All these physical performance tests were performed before a dialysis session. The Asian Working Group for Sarcopenia 2019 and the Chinese Geriatrics Society consensus on Mobility Limitation 2024 recommended SPPB score ≤9 as the cutoff for low physical performance.^23,25^

### Diabetes Assessment

Access to diabetes information was based on the participants' self-reports, and the fasting plasma glucose data were carefully checked again through the electronic medical records; fasting plasma glucose level ≥7.0 mmol/L, 2-hour plasma glucose ≥11.1 mmol/L in an oral glucose tolerance test, or hemoglobin A1c ≥6.5% was considered as diabetes, on the basis of the American Diabetes Association's 2022 recommendations.^26^

### Statistical Analyses

The data with a normal distribution were expressed as mean±SD, whereas the data with a non-normal distribution were expressed as the median, with 25%–75% interquartile ranges given in parentheses. The categorical variables were expressed as proportions. Between-group comparisons were performed using chi-squared test for categorical variables, Mann–Whitney *U* test for skewed continuous variables, and two-sample *t* test for normally distributed continuous variables.

The differences among different SPPB groups were first analyzed. The total population was then divided into diabetic and nondiabetic groups, which were further divided into low-SPPB and high-SPPB subgroups. Afterward, an analysis was made of the differences among low- and high-SPPB groups in diabetic and nondiabetic populations, respectively, with comparisons made among the groups with and without diabetes in low-SPPB and high-SPPB populations. Moreover, comparisons were further made on the differences between diabetic and nondiabetic patients; univariate analyses were performed to determine the correlation of *β*2-MG with SPPB and its component scores, as assessed by Spearman's correlation coefficient.

Binary logistic regression analysis was performed to examine associations of *β*2-MG with physical performance in the nondiabetic and diabetic groups. The interaction effect between *β*2-MG and diabetes was tested by adding the interacted item (*β*2-MG×diabetes) in the logistic regression analysis. Physical performance was used as a dependent variable, and *β*2-MG, as an independent variable. The covariates controlled in the regression models were those with significant between-group differences, which referred to age, sex, hemodialysis duration, BMI, low muscle mass, CVD, smoking, MIS, CCI, hemoglobin, serum albumin, creatinine, BUN, uric acid, calcium, phosphate, iPTH, Kt/V, and URR. The differences of *P* < 0.05 were defined as significant. All statistical analyses were performed with the SPSS V26.0 (SPSS Inc., Chicago, IL).

## Results

**Figure 1:**
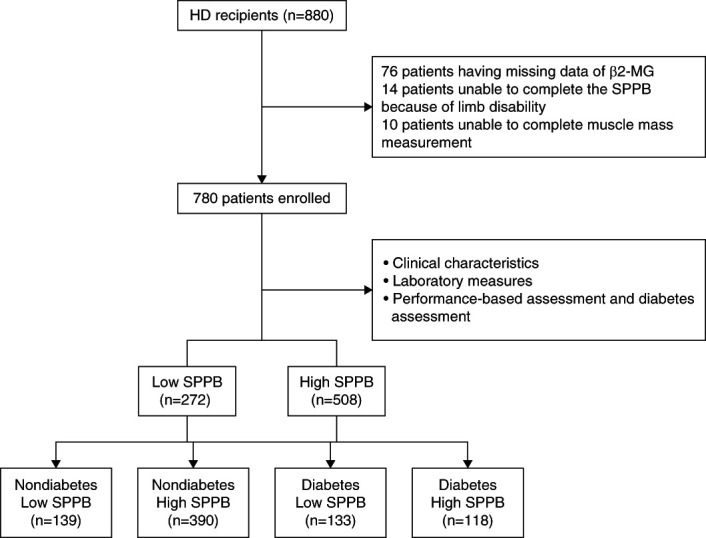
**Flow chart of the study.** *β*2-MG, *β*2-microglobulin; HD, hemodialysis; SPPB, Short Physical Performance Battery.

As shown in Figure 1, a total of 880 patients were enrolled, of whom 100 were excluded: 76 for missing data of *β*2-MG, 14 for being unable to complete the SPPB because of limb disability, and ten for being unable to complete muscle mass measurement. The final analytic sample consisted of 780 participants (male: 482, female: 298; mean age: 61.25 years). As presented in Table 1, the baseline characteristics of the participants were as follows: of all participants, 251 (32.2%) reported diabetes and 272 (34.8%) had low SPPB scores, and the median duration of hemodialysis was 46.92 months, with a maximum of 283.42 months and an interquartile range of 24.54–94.78 months. Low-SPPB patients compared with high-SPPB patients were older and had higher BMI, percentage of low muscle mass, diabetes mellitus and CVD, MIS, and CCI. These patients had shorter hemodialysis duration and lower levels of hemoglobin, serum albumin, BUN, serum creatinine, uric acid, calcium, phosphate, iPTH, and *β*2-MG than high-SPPB patients. No significant differences were observed in terms of Kt/V and URR.

**Table 1:** Differences in clinical characteristics between patients on hemodialysis in different SPPB groups

Characteristic	Total (*n*=780)	Low SPPB (*n*=272)	High SPPB (*n*=508)	*P* Value
Age, yr	61.25±12.67	68.27±11.30	57.50±11.74	<0.001^a^
Male, No. (%)	482 (61.8)	158 (58.1)	324 (63.8)	0.119
Hemodialysis duration, mo	46.92 (24.54–94.78)	43.78 (20.94–76.98)	49.88 (24.71–105.87)	0.046^a^
BMI, kg/m^2^	23.33±3.79	23.78±3.87	23.08±3.73	0.015^a^
Low muscle mass, No. (%)	224 (28.7)	105 (38.6)	119 (23.4)	<0.001^a^
**Comorbidity, No. (%)**				
Hypertension	721 (92.4)	257 (94.5)	464 (92.3)	0.113
DM	251 (32.2)	133 (48.9)	118 (23.2)	<0.001^a^
CVD	194 (24.9)	97 (35.7)	97 (19.1)	<0.001^a^
Smoking, No. (%)	365 (46.8)	122 (44.9)	243 (47.8)	0.426
Drinking, No. (%)	337 (43.2)	111 (40.8)	226 (44.5)	0.323
MIS	4.30±2.80	5.27±3.21	3.78±2.40	<0.001^a^
CCI	3.83±1.66	4.57±1.81	3.44±1.44	<0.001^a^
Hemoglobin, g/L	111.10±15.83	109.56±15.49	111.92±15.96	0.048^a^
Serum albumin, g/L	39.46±3.73	38.84±3.86	39.80±3.61	0.001^a^
Creatinine, *μ*mol/L	985.59±268.36	874.95±239.71	1044.82±264.31	<0.001^a^
BUN, mmol/L	26.46±20.95	24.35±6.25	27.59±25.50	0.039^a^
Uric acid, *μ*mol/L	449.01±92.00	432.28±86.98	458.57±93.51	<0.001^a^
Calcium, mmol/L	2.26±0.25	2.23±0.25	2.27±0.25	0.023^a^
Phosphate, mmol/L	1.95±0.63	1.89±0.67	1.99±0.61	0.034^a^
iPTH, pg/ml	280.10 (143.15–472.53)	268.60 (121.95–428.63)	290.45 (158.18–500.80)	0.017^a^
Total cholesterol, mmol/L	3.90±1.20	3.83±1.16	3.94±1.23	0.218
Triglycerides, mmol/L	2.42±5.09	2.65±8.26	2.30±1.84	0.370
*β*2-MG, mg/L	33.13 (24.14–40.00)	28.83 (20.88–40.00)	34.60 (25.02–40.60)	<0.001^a^
CRP, mg/L	2.77 (1.26–5.89)	2.94 (1.40–5.83)	2.57 (1.17–5.89)	0.394
Kt/V	1.37±0.31	1.36±0.32	1.37±0.30	0.647
URR	67.46±8.40	68.02±7.38	67.14±8.92	0.166

*β*2-MG, *β*2-microglobulin; BMI, body mass index; CCI, Charlson Comorbidity Index; CRP, C-reactive protein; CVD, cardiovascular disease; DM, diabetes mellitus; iPTH, intact parathyroid hormone; MIS, Malnutrition Inflammation Score; SPPB, Short Physical Performance Battery; URR, urea reduction ratio.

a The low SPPB group versus the high SPPB group, *P* < 0.05.

As indicated in Table 2, the low-SPPB group with or without diabetes was more likely to be older, with a higher level of MIS and CCI; a higher percentage of low muscle mass; and a lower level of serum creatinine, BUN, uric acid, and serum albumin than the high-SPPB group. The diabetes group with low or high SPPB scores was prone to have a shorter hemodialysis duration, higher BMI, and higher CCI than the nondiabetes group. In addition, *β*2-MG was lower in the low-SPPB group than in the high-SPPB group for the nondiabetic population, whereas it was lower in the diabetic group than in the nondiabetic group for the high-SPPB population.

**Table 2:** Clinical characteristics classified by diabetes and different SPPB groups (*N*=780)

Characteristic	Nondiabetes (*n*=529)		*P* Value	Diabetes (*n*=251)		*P* Value
	Low SPPB (*n*=139)	High SPPB (*n*=390)		Low SPPB (*n*=133)	High SPPB (*n*=118)	
Age, yr^a^	69.9±11.8	56.7±12.2	<0.001^b^	66.6±10.6^c^	60.1±9.9^d^	<0.001^b^
Male, No. (%)^a^	75 (54.0)	236 (60.5)	0.18	83 (62.4)	88 (74.6)	0.04^b^
Hemodialysis duration, mo^a^	54.9 (28.5–100.4)	57.0 (30.4–118.6)	0.34	37.4 (15.2–59.8)^c^	29.7 (13.8–53.5)^d^	0.16
BMI, kg/m^2^^a^	23.03±3.86	22.74±3.57	0.43	24.56±3.74^c^	24.21±4.02^d^	0.47
Low muscle mass, No. (%)	62 (44.6)	97 (24.9)	<0.001^b^	43 (32.3)^c^	22 (18.6)	0.01^b^
**Comorbidity, No. (%)**						
Hypertension^a^	126 (90.6)	350 (89.7)	0.76	131 (98.5)	114 (96.6)	0.57
CVD^a^	47 (33.8)	61 (15.6)	<0.001^b^	50 (37.6)	36 (30.5)	0.24
Smoking, No. (%)^a^	58 (41.7)	171 (43.8)	0.67	64 (48.1)	72 (61.0)	0.04^b^
Drinking, No. (%)	57 (41.0)	167 (42.8)	0.71	52 (39.1)	58 (49.2)	0.11
MIS^a^	5.10±2.62	3.82±2.42	<0.001^b^	5.45±3.72	3.65±2.32	<0.001^b^
CCI^a^	3.91±1.62	3.03±1.16	<0.001^b^	5.24±1.74^c^	4.77±1.45^d^	0.02^b^
Hemoglobin, g/L^a^	111.1±17.2	112.9±15.7	0.26	107.9±13.4	108.7±16.4^d^	0.67
Serum albumin, g/L	39.1±3.4	39.8±3.6	0.03^b^	38.6±4.3	39.8±3.7	0.03^b^
Creatinine, *μ*mol/L^a^	890.7±218.8	1054.2±263.5	<0.001^b^	858.5±259.6	1030.7±265.8	<0.001^b^
BUN, mmol/L	24.2±6.4	26.4±6.4	0.001^b^	24.5±6.1	27.1±7.0	0.002^b^
Uric acid, *μ*mol/L	434.8±91.5	460.0±95.0	0.01^b^	429.7±82.4	454.0±88.8	0.04^b^
Calcium, mmol/L	2.3±0.3	2.3±0.3	0.99	2.2±0.2^c^	2.3±0.2	0.01^b^
Phosphate, mmol/L	1.8±0.7	1.99±0.6	0.02^b^	1.9±0.6	2.0±0.6	0.41
iPTH, pg/ml^a^	277.5 (104.7–469.1)	312.6 (170.5–541.0)	0.02^b^	242.4 (131.6–381.3)	217.1 (118.7–387.7)^d^	0.32
Total cholesterol, mmol/L	3.8±1.0	3.9±1.3	0.26	3.9±1.3	4.0±1.0	0.55
Triglycerides, mmol/L	3.0±1.4	2.2±1.5	0.38	2.3±2.0	2.8±2.7^d^	0.07
*β*2-MG, mg/L	28.6 (21.5–39.5)	34.8 (25.3–41.4)	<0.001^b^	29.4 (20.7–40.2)	32.3 (24.1–40.0)^d^	0.41
CRP, mg/L	3.2 (1.6–5.9)	2.6 (1.1–5.9)	0.21	2.6 (1.2–5.8)	2.5 (1.2–6.0)	0.83
Kt/V^a^	1.4±0.3	1.4±0.3	0.69	1.3±0.4	1.3±0.3^d^	0.45
URR	68.3±7.5	67.3±9.3	0.26	67.8±7.3	66.6±7.5	0.23

*β*2-MG, *β*2-microglobulin; BMI, body mass index; CCI, Charlson Comorbidity Index; CRP, C-reactive protein; CVD, cardiovascular disease; iPTH, intact parathyroid hormone; MIS, Malnutrition Inflammation Score; SPPB, Short Physical Performance Battery; URR, urea reduction ratio.

a The nondiabetes group (*n*=529) versus the diabetes group (*n*=251), *P* < 0.05.

b In the nondiabetes and diabetes patients, the low SPPB group versus the high SPPB group, *P* < 0.05.

c In the low SPPB patients, the nondiabetes group versus the diabetes group, *P* < 0.05.

d In the high SPPB patients, the nondiabetes group versus the diabetes group, *P* < 0.05.

As shown in Table 3, *β*2-MG was found to be positively associated with SPPB, and its component scores, not in the diabetic population but in the nondiabetic population. The association of *β*2-MG with physical performance was further examined by logistic regression analysis (Table 4). Before the covariates were adjusted, a significant association was observed between *β*2-MG and physical performance in the nondiabetic patients on hemodialysis (odds ratio, 1.034; 95% confidence interval, 1.018 to 1.051; *P* < 0.001) but not in the patients on hemodialysis with diabetes. With the covariates adjusted, these evidences still remained.

**Table 3:** Association of *β*2-microglobulin with SPPB

Variable	Nondiabetes (*n*=529)		Diabetes (*n*=251)	
	*r*	*P* Value	*r*	*P* Value
SPPB score	0.14	0.001^a^	−0.006	0.93
Standing balance test score	0.16	<0.001^a^	−0.005	0.94
5STS test score	0.12	0.006^a^	0.06	0.33
4MGS test score	0.14	0.001^a^	−0.09	0.16

*β*2-MG, *β*2-microglobulin; 4MGS, 4-m gait speed; 5STS, five-repetition sit-to-stand; SPPB, Short Physical Performance Battery.

a In nondiabetes patients, the association of *β2*-microglobulin and short physical performance battery and its components, *P* < 0.05.

**Table 4:** Logistic regression analysis of *β*2-MG and physical performance

Variable	Unadjusted OR (95% CI)	*P* Value	Adjusted Model OR (95% CI)	*P* Value
**Nondiabetes (*n*=529)**				
*β*2-MG	1.034 (1.018 to 1.051)	<0.001^a^	1.040 (1.015 to 1.065)	0.002^a^
**Diabetes (*n*=251)**				
*β*2-MG	1.006 (0.987 to 1.026)	0.55	0.991 (0.961 to 1.020)	0.53
**Interacted item**				
*β*2-MG×diabetes			0.956 (0.923 to 0.990)	0.013^a^

The model was adjusted with age, sex, hemodialysis duration, body mass index, low muscle mass, cardiovascular disease, smoking, Malnutrition Inflammation Score, Charlson Comorbidity Index, hemoglobin, serum albumin, creatinine, BUN, uric acid, calcium, phosphate, intact parathyroid hormone, Kt/V, and urea reduction ratio. *β*2-MG, *β*2-microglobulin; CI, confidence interval; OR, odds ratio.

a In nondiabetes patients, the interacted relationship between *β2*-MG and physical performance, *P* < 0.05.

## Discussion

To the best of our knowledge, this was an inaugural investigation into the potential correlation between *β*2-MG level and physical performance in patients on hemodialysis with and without diabetes. This original research aimed to explore whether elevated *β*2-MG concentrations were associated with diminished physical performance in a cohort of Chinese patients on hemodialysis and to determine whether this association differed in the presence of diabetes. In our study, however, the findings were not entirely consistent with our original premise. In an assessment conducted using the SPPB, we uncovered a notably positive association between *β*2-MG and physical function in patients on hemodialysis devoid of diabetes. This significant correlation, however, was not observed in patients on hemodialysis with diabetes.

Intriguingly, the discovery of a positive correlation between *β*2-MG and SPPB was an unexpected finding. One previous study reported that the levels of plasma NT-pro-terminal B-type natriuretic peptide, albumin, and hemoglobin were associated with physical performance; however, the research did not investigate the relation of *β*2-MG with physical function.^27^ Another study delved into the correlations between serum uremic toxins, including *β*2-MG, and declining physical performance in patients on dialysis.^28^ We found that these reported evidences did not reveal a consistent association between *β*2-MG levels and various indicators of physical capability, which included the six-minute walk test, the ten-repetition sit-to-stand test, handgrip strength measurements, and scores from the Human Activity Profile questionnaire.

The rationale behind our findings remains elusive, considering that *β*2-MG facilitates antigen presentation and is considered a marker for the activation of the cellular immune system presentation.^4^ Korean studies have indicated that higher serum *β*2-MG levels are associated with better survival in patients on dialysis.^13,14^ One study reported a hypothesis that patients with extremely low *β*2-MG levels developed impaired immunity and that malnutrition could suppress the overall immune system, resulting in suppression of *β*2-MG production.^13^ Actually, the categorization of *β*2-MG levels has varied across different studies. In our research, the median *β*2-MG level was recorded at 33.20 mg/L, reaching a maximum of 67.8 mg/L. It is conceivable that there would exist a specific range within which *β*2-MG exerts a beneficial effect; however, this hypothesis warrants validation through further investigation with a larger sample size.

*β*2-MG, like BUN, serum creatinine, and uric acid, is a representative uremic marker. *β*2-MG was previously reported to reflect nutritional status, as in the case of creatinine, BUN, and uric acid, and might be likely to take part as the markers of reverse epidemiology.^14^ According to some previous studies, higher BUN and serum creatinine indicate better nutritional status, hence a higher survival rate in patients on dialysis.^29–31^ It was comfirmed that the association of low uric acid with higher mortality was explained by the association of low uric acid with both sarcopenia and pathologic weight loss.^32^ In our study, the high-SPPB group had a higher level of BUN and creatinine than the low-SPPB group (but with an equal level of Kt/V between them), which may have been ascribed to the better nutritional status. Elevated serum creatinine was once reported to function as an indication of increased muscle mass or meat ingestion; therefore, the patients on dialysis with higher serum creatinine lived longer.^31^ Similarly, we have come to believe that patients on hemodialysis with better nutritional status live longer, extending their duration of dialysis.^14^ In this study, moreover, the low-SPPB group had a higher level of CCI than the high-SPPB group in patients on hemodialysis with or without diabetes, which suggested the association of significant comorbidity with diminished physical performance. In view of the above, we can hypothesize that serum *β*2-MG concentration could be elevated in those who undergo hemodialysis for a longer duration, which could be ascribed to better nutritional health.

Consistent with previous studies,^33,34^ our study confirmed that patients on hemodialysis with diabetes had lower SPPB scores. Diabetes may affect muscle function through some mechanisms, as described by the previous evidence that persistent hyperglycemia could contribute to the longitudinal decline in muscle mass and strength.^35,36^ In the diabetic patients, poor glycemic control was found to lead to low muscle mass, and a proposal was put forward that the association between hyperglycemia and low muscle mass might be bidirectional.^37^ The accumulation of advanced glycation end products and impaired insulin action are considered as potential underlying factors that may elucidate this association.^38^ Diabetic patients may have macrovascular and microvascular complications, such as stroke, coronary arterial disease, neuropathy, peripheral vascular disease, diabetic nephropathy, retinopathy, and neuropathy, which all can decrease physical activity and cause decreased physical function in the dialysis population.^39,40^ Antidiabetic medications have also been identified to have potential negative effects on muscle mass and performance.^41^ In this study, however, we did not find a correlation between *β*2-MG and physical fitness in the hemodialysis population with diabetes, probably because of the strong inverse correlation between diabetes and physical function. Furthermore, elevated glucose concentration was demonstrated to be positively associated with elevated *β*2-MG concentration in patients on dialysis.^20^ This could potentially obscure or weaken the positive correlation between *β*2-MG and physical fitness, specifically within the diabetic subset of the dialysis population. Understanding these intricate relationships is crucial for the development of comprehensive care strategies to improve their muscle function and overall quality of life in patients on hemodialysis with diabetes.

In addition, we identified the evidence that there was a higher BMI in the low-SPPB and high-SPPB groups, formed based on the total population of participants. Further division indicated no difference in BMI between the low- and high-SPPB groups with or without diabetes. Of note was the evidence that the low-SPPB group had more patients with low muscle mass, regardless of the presence of diabetes, than the high-SPPB group. Previous studies have shown that as BMI rises, the number of diabetic patients and the administration of diabetic medication increases,^42^ and that lower BMI is significantly associated with sarcopenia.^43^ This tendency can persist irrespective of diabetes status, suggesting that the patients who have lower scores for physical performance are likely to have a higher fat composition and sarcopenia, despite a BMI that does not categorically suggest undernutrition. This phenomenon underscores the potential for sarcopenic obesity—a condition where individuals exhibit both excess body fat and diminished muscle mass. The underlying cause may be attributed to the effects of volume overload. In addition, we believe BMI may be a mediating factor in diabetes and low muscle mass.

In this multicenter investigation, there were several limitations for discussion. Despite careful adjustment for numerous potential confounders, there may have still been additional factors that were not accounted for, which could influence the outcomes. The unmeasured variables could introduce bias or alter the associations observed. Next, the residual renal function was not thoroughly assessed in our study. The study cohort had a median hemodialysis duration of 46.92 months (the longest being up to 283.42 months), with an interquartile range from 24.54-94.78 months, which suggested that many patients may have possessed minimal residual renal function. An in-depth evaluation of residual kidney function could provide further clarity on its effect on the associations investigated. In addition, given the cross-sectional nature of our study, we were unable to infer causality from the associations observed. The directionality of the associations and the possibility of reverse causation cannot be ascertained with this design. In our future research, it is imperative that we develop a longitudinal study design on the basis of a large sample size.

Our retrospective observational results confirmed the relation between *β*2-MG and SPPB varied with Chinese patients on hemodialysis with and without diabetes. A lower level of *β*2-MG was significantly associated with poor physical performance in patients on hemodialysis without diabetes, which is necessary for nephrologists to take into account. The implications of *β*2-MG seem to be complex and multifaceted, suggesting the clinical relevance of this marker broader than previously understood. Further studies need to be conducted to explore the mechanisms so as to confirm the significance of *β*2-MG across different populations and comorbidities.

## Supplementary Material

SUPPLEMENTARY MATERIAL

## Data Availability

Partial restrictions to the data and/or materials apply. The datasets used and/or analyzed during the current study are available from the corresponding author upon reasonable request.
