# Supplementary material for: Association of β2-Microglobulin with Physical Performance in Chinese Hemodialysis Patients with and without Diabetes
Source: Kidney360. 2024 Dec 3;6(3):412–9. doi: 10.34067/KID.0000000669 (PMC11970847; doi:10.34067/KID.0000000669)
Supplement: SUPPLEMENTARY MATERIAL [file kidney360-6-412-s001.pdf]

ASN American Society of Nephrology
JASN Journal of the American Society of Nephrology
KIDNEY360 Accessing Our World From Every Angle
CJASN Clinical Journal of the American Society of Nephrology

ASN Journal Disclosure Form

As per ASN journal policy, I have disclosed any financial relationships or commitments I have held in the past 36 months as included below. I have listed my Current Employer below to indicate there is a relationship requiring disclosure. If no relationship exists, my Current Employer is not listed.

W. Chu has nothing to disclose.

I understand that the information above will be published within the journal article, if accepted, and that failure to comply and/or to accurately and completely report the potential financial conflicts of interest could lead to the following: 1) Prior to publication, article rejection, or 2) Post-publication, sanctions ranging from, but not limited to, issuing a correction, reporting the inaccurate information to the authors' institution, banning authors from submitting work to ASN journals for varying lengths of time, and/or retraction of the published work.

Name: Wenwen Chu
Manuscript ID: K360-2024-000604R1
Manuscript Title: The association of β2-Microglobulin with physical performance in Chinese hemodialysis patients with and without diabetes
Date of Completion: November 5, 2024
Disclosure Updated Date: November 5, 2024

ASN American Society of Nephrology | JASN Journal of the American Society of Nephrology | KIDNEY360 Accessing Our World From Every Angle | CJASN Clinical Journal of the American Society of Nephrology

ASN Journal Disclosure Form

As per ASN journal policy, I have disclosed any financial relationships or commitments I have held in the past 36 months as included below. I have listed my Current Employer below to indicate there is a relationship requiring disclosure. If no relationship exists, my Current Employer is not listed.

Q. Guo has nothing to disclose.

I understand that the information above will be published within the journal article, if accepted, and that failure to comply and/or to accurately and completely report the potential financial conflicts of interest could lead to the following: 1) Prior to publication, article rejection, or 2) Post-publication, sanctions ranging from, but not limited to, issuing a correction, reporting the inaccurate information to the authors' institution, banning authors from submitting work to ASN journals for varying lengths of time, and/or retraction of the published work.

Name: Qi Guo
Manuscript ID: K360-2024-000604R1
Manuscript Title: The association of β2-Microglobulin with physical performance in Chinese hemodialysis patients with and without diabetes
Date of Completion: November 6, 2024
Disclosure Updated Date: November 6, 2024

ASN American Society of Nephrology
JASN Journal of the American Society of Nephrology
KIDNEY360 Accessing Our World From Every Angle
CJASN Clinical Journal of the American Society of Nephrology

ASN Journal Disclosure Form

As per ASN journal policy, I have disclosed any financial relationships or commitments I have held in the past 36 months as included below. I have listed my Current Employer below to indicate there is a relationship requiring disclosure. If no relationship exists, my Current Employer is not listed.

Q. Zhan reports the following:
Employer: Shanghai University of Medicine and Health Sciences Affiliated Zhoupu Hospital

I understand that the information above will be published within the journal article, if accepted, and that failure to comply and/or to accurately and completely report the potential financial conflicts of interest could lead to the following: 1) Prior to publication, article rejection, or 2) Post-publication, sanctions ranging from, but not limited to, issuing a correction, reporting the inaccurate information to the authors' institution, banning authors from submitting work to ASN journals for varying lengths of time, and/or retraction of the published work.

Name: Qiunan Zhan
Manuscript ID: K360-2024-000604R1
Manuscript Title: The association of β2-Microglobulin with physical performance in Chinese hemodialysis patients with and without diabetes
Date of Completion: November 4, 2024
Disclosure Updated Date: November 4, 2024

ASN American Society of Nephrology JASN Journal of the American Society of Nephrology KIDNEY360 Accessing Our World From Every Angle CJASN Clinical Journal of the American Society of Nephrology

ASN Journal Disclosure Form

As per ASN journal policy, I have disclosed any financial relationships or commitments I have held in the past 36 months as included below. I have listed my Current Employer below to indicate there is a relationship requiring disclosure. If no relationship exists, my Current Employer is not listed.

J. Zhao has nothing to disclose.

I understand that the information above will be published within the journal article, if accepted, and that failure to comply and/or to accurately and completely report the potential financial conflicts of interest could lead to the following: 1) Prior to publication, article rejection, or 2) Post-publication, sanctions ranging from, but not limited to, issuing a correction, reporting the inaccurate information to the authors' institution, banning authors from submitting work to ASN journals for varying lengths of time, and/or retraction of the published work.

Name: Junli Zhao
Manuscript ID: K360-2024-000604R1
Manuscript Title: The association of β2-Microglobulin with physical performance in Chinese hemodialysis patients with and without diabetes.
Date of Completion: November 4, 2024
Disclosure Updated Date: November 4, 2024
